# The Influence of Music on Mental Health Through Neuroplasticity: Mechanisms, Clinical Implications, and Contextual Perspectives

**DOI:** 10.3390/brainsci15111248

**Published:** 2025-11-20

**Authors:** Yoshihiro Noda, Takahiro Noda

**Affiliations:** 1 Department of Psychiatry, International University of Health and Welfare, Mita Hospital, Tokyo 108-8329, Japan; 2 Graduate School of Medical Sciences, Kyushu University, Fukuoka 812-8582, Japan

**Keywords:** music, neuroplasticity, mechanisms, music therapy, mental health, translational recommendations

## Abstract

Music is a near-universal anthropological and sensory phenomenon that engages distributed brain networks and peripheral physiological systems to shape emotion, cognition, sociality, and bodily regulation. Evidence from electrophysiology, neuroimaging, endocrinology, randomized controlled trials, and longitudinal training studies indicates that both receptive and active musical experiences produce experience-dependent neural and systemic adaptations. These include entrainment of neural oscillations, modulation of predictive and reward signaling, autonomic and neuroendocrine changes, and long-term structural connectivity alterations that support affect regulation, cognition, social functioning, motor control, sleep, and resilience to neuropsychiatric illness. This narrative review integrates mechanistic domains with clinical outcomes across major conditions, such as depression, anxiety, schizophrenia, dementia, and selected neurodevelopmental disorders, by mapping acoustic and procedural parameters onto plausible biological pathways. We summarize how tempo, beat regularity, timbre and spectral content, predictability, active versus passive engagement, social context, dose, and timing influence neural entrainment, synaptic and network plasticity, reward and prediction-error dynamics, autonomic balance, and immune/endocrine mediators. For each condition, we synthesize randomized and observational findings and explicitly link observed improvements to mechanistic pathways. We identify methodological limitations, including heterogeneous interventions, small and biased samples, sparse longitudinal imaging and standardized physiological endpoints, and inconsistent acoustic reporting, and translate these into recommendations for translational trials: harmonized acoustic reporting, pre-specified mechanistic endpoints (neuroimaging, autonomic, neuroendocrine, immune markers), adequately powered randomized designs with active controls, and long-term follow-up. Contextual moderators including music education, socioeconomic and cultural factors, sport, sleep, and ritual practices are emphasized as critical determinants of implementation and effectiveness.

## 1. Introduction

Music is both an ancient cultural practice and a structured sensory stimulus that reliably engages distributed brain networks and peripheral physiological systems [1,2]. Across cultures and historical epochs, musical participation has served as a vehicle for ritual, communication, education, and emotional expression [3,4]. Contemporary neuroscience now provides converging evidence that these practices extend beyond transient affect regulation to induce lasting functional and structural brain changes [2,5]. Findings from electrophysiology, neuroimaging, endocrinology, randomized controlled trials, and longitudinal training studies demonstrate that musical experience, whether receptive or active, can shape neural oscillations, predictive and reward dynamics, autonomic and neuroendocrine regulation, and long-term connectivity, thereby supporting cognition, social functioning, motor control, sleep, and resilience to neuropsychiatric illness [6,7,8,9,10,11].

This review pursues a focused aim: to synthesize mechanistic evidence that links acoustic properties and modes of musical engagement to experience-dependent neuroplasticity, and to map these mechanisms onto clinical outcomes in major neuropsychiatric disorders, including depression, anxiety, schizophrenia, dementia, and selected neurodevelopmental conditions [1,2]. Music is simultaneously an organized acoustic signal and a socially embedded practice [3,4]. As an acoustic signal, it provides temporally structured variations in frequency, intensity, and spectral content, which the nervous system processes through hierarchical predictive and sensorimotor mechanisms [6,12,13]. As a social practice, repeated musical participation links affective salience, interpersonal synchrony, and reward, which are conditions favorable to plastic change [7,8,9]. The convergence of these properties explains why music produces immediate subjective effects, such as affect modulation and arousal shifts, and, with repeated exposure or training, durable neural adaptations that can underpin clinical improvement and long-term resilience [2,5,10,11].

In addition to mechanistic pathways, contextual dimensions, including cultural and socioeconomic factors, education, sleep, sport, and spiritual or ritual practices, are considered as moderators of implementation and translational potential [3,14,15]. These domains do not serve as primary mechanistic drivers but critically shape accessibility, adherence, and cultural adaptability of interventions [4,15]. By integrating evidence across scales, from molecular and neurochemical processes to neural dynamics, structural connectivity, and behavior, this review aims to provide pragmatic guidance for the design of future interventions and mechanistic, hypothesis-driven clinical trials [2,16]. Thus, music is framed not only as a universal cultural artifact but also as a dynamic agent fostering neuroplasticity and promoting mental health [2,3].

## 2. Methods

### 2.1. Narrative Approach and Selection Criteria

This review employed a targeted, mechanism-focused narrative synthesis rather than a comprehensive systematic survey [1,2]. Our methodological approach drew upon recent primary studies, meta-analyses, and relevant reviews across neuroscience, clinical psychiatry, psychology, and ethnomusicology [15,16]. The central objective was to integrate mechanistic evidence linking specific acoustic and procedural parameters of musical experience to neurophysiological and clinical outcomes, and to highlight research designs capable of supporting causal inference [12,17].

Priority was given to three categories of evidence. First, we examined experimental and longitudinal neuroscience studies utilizing electroencephalography (EEG), magnetoencephalography (MEG), functional magnetic resonance imaging (fMRI), diffusion tensor imaging (DTI), and positron emission tomography (PET) that explicitly connected acoustic features or modes of engagement to neurophysiological endpoints, including neural oscillations, connectivity, and structural plasticity [5,6,13]. Second, we incorporated randomized controlled trials (RCTs) and meta-analyses that reported clinical outcomes in major neuropsychiatric conditions such as depression, anxiety, schizophrenia, dementia, and selected neurodevelopmental disorders [7,8,11]. Third, we reviewed systematic analyses and conceptual frameworks that bridge mechanistic neuroscience with ethnomusicological and implementation perspectives, thereby situating music within broader cultural and translational contexts [15,16].

Where authoritative systematic reviews or Cochrane analyses existed for specific clinical conditions, their conclusions were integrated to strengthen the evidentiary base [18,19,20]. Longitudinal imaging studies documenting structural or connectivity changes following music training or therapy were given particular emphasis, as they provide critical insight into experience-dependent neuroplasticity [2,5,12].

The goal of this synthesis was not exhaustive bibliographic coverage, but rather the clarification of mechanistic pathways and the identification of methodological strategies most capable of linking acoustic and procedural parameters to neurophysiological endpoints and clinical outcomes [15,16]. By integrating evidence across disciplinary boundaries and methodological scales, this review aims to provide pragmatic guidance for the design of future mechanistic, hypothesis-driven clinical trials and to inform the translational development of music-based interventions [2,16].

### 2.2. Minimum Acoustic and Procedural Reporting Checklist

• Intervention label: descriptive name; active/receptive; group/individual.
• Tempo: beats per minute (bpm) or range; method used to set tempo.
• Beat regularity: isochronous/polyrhythmic/variable (specify).
• Meter and time signature: notation or description (e.g., 4/4, free meter).
• Session dose: session duration (min), sessions per week, total intervention weeks.
• Engagement mode: active (playing/singing/improvisation) or passive (listening); degree of instruction.
• Sound level: mean and range in dB(A) and/or root mean square (RMS) when relevant; measurement device and distance.
• Timbre descriptors: instrument(s), vocal/instrumental mix, spectral descriptors (e.g., narrow/wide bandwidth; harmonic/inharmonic).
• Predictability/novelty: annotated expectancy violations, entropy index, or short descriptor (e.g., repeated scaffolded melodies vs. highly novel stimuli).
• Personalization/preference: whether repertoire was individualized; measure of participant preference and its use in selection.
• Delivery format and setting: live vs. recorded; in-person vs. remote/digital; acoustic environment (room acoustics if applicable).
• Control condition description: contents of control or comparator (e.g., active control music, white noise, silence, non-musical social activity).
• Adherence and fidelity metrics: attendance, practice logs, objective usage data (if digital), and fidelity checks for facilitator training.
• Timing of mechanistic sampling: exact timing of physiological/neuroimaging sampling relative to sessions (pre/post; minutes/hours).

Operationalizing these priorities will reduce duplication, strengthen causal inference, and accelerate translation while preserving cultural adaptability and equity.

## 3. Results

### 3.1. Mechanistic Pathways of Music and Neuroplasticity

Our synthesis identified six interrelated mechanistic domains through which music exerts neuroplastic effects. These domains interact dynamically, and each can be selectively engaged by manipulating acoustic and procedural parameters. Together, they form a cascade from acoustic input to neural entrainment, predictive and reward dynamics, oscillatory modulation, systemic mediators, structural adaptation, and behavioral reinforcement, ultimately shaping clinical outcomes [1,2,5,6,7,8,9,10,13,17].

#### 3.1.1. Acoustic Determinants and Neural Entrainment (See Section 2.2)

Key acoustic features, tempo, beat regularity, timbre (spectral composition), loudness dynamics, and predictability, determine the degree and specificity of neural entrainment [21,22]. Regular beats and tempo matching to physiological rhythms promote phase-locking of cortical and subcortical oscillations, improving temporal prediction and sensorimotor coordination [13,17]. For example, slow tempos (~60–80 beats per minute (bpm)) facilitate parasympathetic activation and sleep onset, while tempos matched to gait cadence optimize motor entrainment in rehabilitation [9,23]. Predictability versus controlled surprise governs prediction-error signaling; moderate, context-constrained surprise maximizes hedonic responses and dopaminergic reinforcement [6,17].

Practical implications: Intervention design should precisely report and manipulate tempo (bpm), beat regularity (isochronous vs. polyrhythmic), timbre descriptors (bandwidth, harmonicity), loudness contours (dB range, root mean square), and indices of predictability (entropy measures, annotated expectancy violations) [2,24].

#### 3.1.2. Predictive Coding, Reward, and Large-Scale Network Dynamics

Listening and performing music activate auditory, prefrontal, motor, limbic, and striatal networks [1,12]. Predictive coding models explain how expectancy violations generate reward responses: anticipation and consummatory phases recruit distinct dopaminergic pathways [6,25]. Enhanced connectivity between auditory regions and the nucleus accumbens/ventral tegmental area supports motivation and reinforcement, biasing plasticity in target networks [1,6]. Active performance amplifies auditory–motor coupling and strengthens links between auditory cortex, prefrontal control regions, limbic structures, and striatal reward circuits [12,13].

Practical implications: Combine predictable scaffolding with controlled novelty in intervention stimuli; incorporate active tasks (e.g., improvisation, call-and-response) to engage reward and motor networks [6,17].

#### 3.1.3. Oscillatory Entrainment and Cognitive Modulation

Rhythmic structure entrains canonical frequency bands with distinct cognitive correlates [21,26]. Slow, calming music increases alpha and theta activity associated with relaxation and memory consolidation; groove-oriented or faster rhythms elevate beta and gamma activity linked to attention and sensorimotor integration [1,27]. Sustained entrainment stabilizes cross-regional coupling (frontal–temporal–motor), supporting working memory, temporal prediction, and attentional control [12,13]. Passive listening predominantly modulates sensory and limbic dynamics, whereas active performance engages motor cortex and cerebellum more robustly [2,5].

Practical implications: Select rhythm-tonal features aligned with cognitive targets (e.g., slow tempo for sleep, groove for attention/motor tasks) and measure oscillatory endpoints (EEG/MEG) to test hypothesized mediations [21,27].

#### 3.1.4. Autonomic, Neuroendocrine, and Immune Mediators

Music modulates autonomic balance, neuroendocrine secretion, and immune function [9,10]. Calming, preferred music increases parasympathetic activity (heart-rate variability), lowers heart rate and blood pressure, and reduces cortisol [9,10]. Social music-making elevates oxytocin and endogenous opioids, and in some contexts reduces inflammatory markers [28,29]. These systemic shifts create a biochemical environment conducive to synaptic plasticity by decreasing stress-related inhibition and enhancing neuromodulatory support for consolidation [30,31].

Practical implications: Trials should include autonomic (heart rate variability: HRV), endocrine (cortisol, oxytocin), and immune markers as pre-specified mechanistic endpoints, with careful attention to timing of sampling relative to intervention [29,32].

#### 3.1.5. Structural Connectivity and Experience-Dependent Plasticity

Longitudinal MRI and DTI studies demonstrate experience-dependent changes in white-matter tracts (e.g., arcuate fasciculus, corpus callosum) and gray-matter volume in auditory, motor, and prefrontal regions following prolonged training [2,5,33,34]. Sensitive periods amplify developmental effects, but adult neuroplasticity remains demonstrable with sufficient intensity and duration of practice or structured therapy [2,35]. Functional reorganization and connectivity changes are evident even in mature brains, underscoring music’s potential as a lifelong catalyst for plasticity [36,37].

Practical implications: Integrate pre- and post-intervention imaging (DTI, structural MRI) in longitudinal trials, report effect sizes and reproducibility across cohorts, and harmonize acquisition/analysis parameters to strengthen causal attribution [2,5].

#### 3.1.6. Behavioral and Social Reinforcement: Mechanisms of Metaplasticity

Active, socially embedded musical practice uniquely combines sensorimotor demands with social bonding and reward [28,38]. Group activities (ensemble playing, choirs, drumming circles) promote oxytocinergic bonding, synchronize physiological and neural states, and provide repeated, salient reinforcement that can induce metaplastic modulation, altering the propensity for subsequent plasticity [29,32]. These processes enhance adherence, amplify hedonic salience, and consolidate practice-dependent neural changes [28,38].

Practical implications: Design interventions with repeated, socially reinforced engagement, and include ecological measures of adherence, synchrony, and group cohesion to capture metaplastic effects [29,38].

#### 3.1.7. Mechanistic Linkage Summary

**Figure 1:**
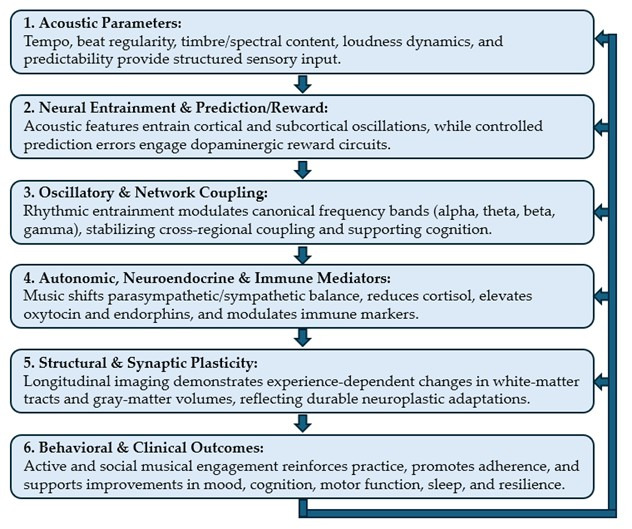
Mechanistic Cascade Relationships between Music and Neuroplasticity.

Across domains, the evidence supports a cascading model (see Figure 1 below): Acoustic parameters → neural entrainment and prediction/reward → oscillatory and network coupling → autonomic/neurochemical environment → synaptic and structural plasticity → behavioral and clinical outcomes [1,2,5,6,7,8,9,10,17]. This framework highlights how carefully designed musical interventions can harness multisystem mechanisms to promote neuroplasticity, resilience, and mental health [2,6,17].

This schematic illustrates the six interconnected domains through which music drives neuroplasticity and clinical outcomes. Flow: Arrows indicate a cascading sequence: Acoustic input → Neural entrainment and reward → Oscillatory coupling → Autonomic/neurochemical modulation → Structural/synaptic plasticity → Behavioral and clinical outcomes. Feedback loops highlight bidirectional reinforcement, particularly between social engagement, reward circuitry, and adherence.

### 3.2. Clinical Applications and Mechanistic Linkages

The clinical literature on music-based interventions demonstrates promising benefits across multiple neuropsychiatric and neurological conditions, yet remains heterogeneous in intervention type, dose, setting, and outcome measures. By synthesizing evidence from RCTs, meta-analyses, and systematic reviews, and mapping these findings onto mechanistic pathways, we highlight both the therapeutic potential of music and the methodological challenges that must be addressed to advance the field [1,2].

#### 3.2.1. Depression

Evidence: Multiple RCTs and meta-analyses consistently support adjunctive music therapy for reducing depressive symptoms and improving mood and social functioning [11,18,39]. Benefits are observed across diverse populations, though heterogeneity in protocols and small sample sizes limit precision regarding effect sizes and moderators [1,2].

Mechanistic linkage: Music engages auditory-reward circuits, reducing anhedonia through dopaminergic signaling [6], enhances amygdala–prefrontal coupling to support affect regulation, and fosters social bonding via oxytocinergic pathways in group formats [28]. Repeated rewarding experiences may bias connectivity toward more adaptive emotion-regulation networks [2,5].

Research priorities: Adequately powered multicenter RCTs specifying acoustic parameters and engagement formats, inclusion of mechanistic endpoints (EEG markers of reward anticipation, fMRI connectivity of affect networks, HRV/cortisol) [9,10], and mediation analyses to test whether reward-system engagement predicts clinical change [6,11].

#### 3.2.2. Anxiety Disorders and Procedural Stress

Evidence: Short music interventions reliably reduce state anxiety and physiological arousal in perioperative and acute procedural contexts. Evidence for chronic anxiety disorders is smaller but promising [20,40].

Mechanistic linkage: Slow tempo and regular rhythmic features facilitate parasympathetic activation, reduce sympathetic tone, and modulate amygdala–prefrontal coupling, thereby lowering subjective and physiological anxiety. Reduced cortisol further supports plasticity favoring improved stress reactivity [9,10].

Research priorities: Trials examining dose–response relationships in chronic anxiety, inclusion of objective physiological endpoints (HRV, cortisol), and long-term follow-up to assess durability of resilience [10,20,40].

#### 3.2.3. Neurodevelopmental Disorders (ASD and ADHD)

Evidence: Improvisational and structured music therapies improve joint attention, imitation, and social reciprocity in ASD, while rhythm-based training enhances attentional control in ADHD. Although sample sizes are often small, systematic reviews indicate consistent directionality of effects [41,42,43,44].

Mechanistic linkage: Rhythm scaffolds temporal prediction, turn-taking, and sensorimotor synchrony, entraining motor and attentional networks [23,45]. Social musical interaction enhances oxytocinergic facilitation of social learning [28,30]. Early, repeated training may leverage sensitive periods, producing lasting developmental effects [46].

Research priorities: Longitudinal, adequately powered trials beginning in early childhood, combining behavioral endpoints with developmental neuroimaging (DTI, functional connectivity), and accounting for heterogeneity within diagnostic groups [42,43,47].

#### 3.2.4. Schizophrenia

Evidence: Group and individual music therapy consistently improve negative symptoms (social withdrawal, affective flattening), social functioning, and quality of life, while effects on positive symptoms are limited [48,49,50,51].

Mechanistic linkage: Nonconfrontational musical engagement activates reward circuitry, reduces stress physiology, and strengthens affective and interpersonal networks. These processes may improve social cognition and behavioral activation through changes in functional connectivity of social brain networks [15,52].

Research priorities: Mechanistic trials testing whether changes in reward circuitry or social-cognitive networks mediate symptom improvement, and whether intervention characteristics (group vs. individual; active vs. receptive) moderate outcomes [48,49,53].

#### 3.2.5. Dementia and Mild Cognitive Impairment

Evidence: Individualized familiar-music interventions and group singing reliably reduce agitation and improve engagement. Transient cognitive benefits for verbal fluency and attention have been observed, though evidence for disease-modifying structural change remains limited [19,54,55].

Mechanistic linkage: Familiar music engages preserved limbic and autobiographical memory circuits (amygdala–hippocampus), stabilizes autonomic arousal and mood, and promotes social engagement. Repeated activity may help maintain functional networks underpinning affect and behavior despite progressive pathology [7,56,57].

Research priorities: RCTs with adequately powered samples assessing both symptomatic and mechanistic endpoints (EEG/fMRI responses to familiar music, autonomic measures), and longer follow-up to examine durability and potential slowing of functional decline [7,19,58].

#### 3.2.6. Sleep and Motor Rehabilitation

Evidence: Slow-tempo pre-sleep music improves subjective sleep quality and reduces sleep latency in insomnia samples [59,60,61]. Rhythmic auditory stimulation yields robust gait improvements in Parkinson’s disease and benefits in stroke rehabilitation [7,8,62,63].

Mechanistic linkage: Tempo and spectral content entrain physiological and neural rhythms supportive of sleep onset or optimized motor timing. For gait rehabilitation, auditory–motor coupling facilitates entrainment and relearning of motor sequences.

Research priorities: Dose-optimization studies (duration, frequency), mechanistic endpoints linking oscillatory changes to sleep architecture (polysomnography/EEG), and integration with wearable sensors to measure real-world effectiveness [62,64,65].

#### 3.2.7. Overall Statement

Across domains the strongest randomized evidence exists for symptomatic benefit (depression adjunctive therapy; peri-procedural anxiety reduction; motor improvements with rhythmic auditory stimulation) [8,11,20,62], but mechanistic linkage to durable neuroplastic change remains uneven [2,5,6]. Common cross-cutting limitations are small, often single-site trials; heterogeneity and incomplete reporting of acoustic and procedural parameters; infrequent pre-specified mechanistic endpoints (EEG, fMRI, DTI, HRV, endocrine markers); incomplete blinding or active control conditions; and short follow-up durations [18,19,40,65]. These constraints informed our evidence-grading judgments in the table below and motivated the explicit research priorities recommended throughout the revised manuscript. Table 1 below shows the Overview of Evaluation Summaries on the Effects of Music Therapy for Major Mental Health Disorders [48,66].

### 3.3. Integrative Perspective

**Figure 2:**
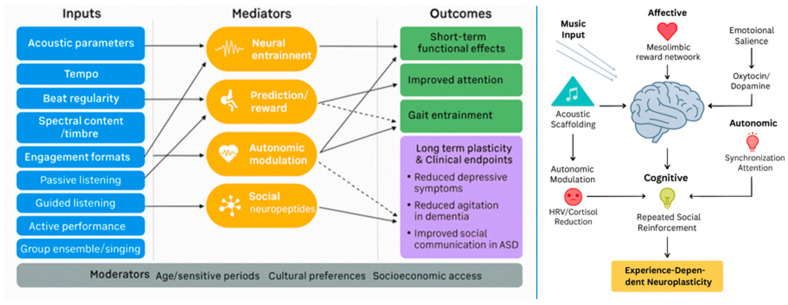
Multisystemic mechanisms linking musical inputs to neuroplastic and mental health outcomes.

A schematic overview (Figure 2) illustrating the effects of musical stimulation on mental health mediated through neuroplasticity is shown below. Across conditions, music’s therapeutic effects appear to arise from convergent multisystem mechanisms: acoustic entrainment of neural oscillations [2,70], modulation of reward and prediction-error signaling [6], stabilization of autonomic and neuroendocrine balance [9,10], and reinforcement through social engagement [7,70]. These mechanisms collectively support synaptic and structural plasticity, which in turn underpin improvements in mood, cognition, motor function, sleep, and resilience [5,7,66].

The field now requires harmonization of intervention protocols, standardized reporting of acoustic parameters, and incorporation of mechanistic endpoints into clinical trials [40,65]. Longitudinal designs with active controls and sufficient power are essential to establish causal pathways and durability of effects [5,18]. By integrating mechanistic neuroscience with clinical psychiatry, psychology, and ethnomusicology, future research may move beyond symptomatic improvement to targeted, hypothesis-driven interventions that harness music as a scalable, culturally adaptable catalyst for neuroplasticity and mental health [2,66].

Schematic overview of proposed multisystemic mechanisms by which music (singing, rhythmic auditory simulation, instrumental, or listening) elicits experience-dependent neuroplasticity. Note for the left panel: solid line represents relationships supported by stronger longitudinal or interventional evidence (e.g., randomized controlled trials, well-controlled longitudinal cohorts) that provide temporal ordering and greater inferential confidence regarding association or causal direction; dashed line represents relationships supported primarily by cross-sectional, correlational, or limited preliminary evidence (e.g., small observational studies, single-site reports, or indirect mechanistic inferences); these pathways are presented as provisional or hypothesis-generating and require longitudinal or experimental validation.

### 3.4. Contextual Perspectives and Implementation Considerations

The structured, evidence-informed deployment of music-based interventions requires attention not only to proximal mechanistic targets but also to the broader cultural, social, temporal, and delivery ecosystems that ultimately determine uptake, efficacy, equity, and sustainability [15,16,71]. Drawing on anthropological, educational, sleep, motor, and spiritual perspectives, the following discussion integrates mechanistic insights with pragmatic considerations to guide translational design, evaluation, and scale-up across prevention, rehabilitation, and public-health domains [15,16,38,71,72,73].

#### 3.4.1. Music Education as a Public Health Lever

Formal and community music education provides a durable platform for repeated, salient practice episodes that foster structural and functional plasticity across the lifespan [2,5,66]. When reframed with explicit therapeutic aims, educational settings may deliver preventive cognitive benefits and rehabilitative gains at scale [7,72]. To achieve this, curricula must embed measurable clinical and functional targets, such as improvements in working memory, emotional regulation, or gait timing, and establish fidelity metrics for dosage, practice structure, and instructor training [15,16,71].

Economic evaluation is equally critical, requiring assessment of cost-effectiveness, funding pathways, and mechanisms to mitigate socioeconomic barriers to participation [74,75]. Importantly, sensitive-period interventions in childhood should be complemented by lifelong learning opportunities for adults and older adults, thereby maximizing developmental plasticity while supporting maintenance and remediation [2,74]. Embedding therapeutic aims into longstanding educational infrastructures leverages music’s role in identity formation and social cohesion, while preserving cultural specificity and community ownership [38,75,76].

#### 3.4.2. Socioeconomic and Cultural Moderators

Music’s functions and effects are deeply shaped by historical, ecological, and cultural variation. Repertoires, timbres, ensemble formats, and ritual roles differ across regions and social contexts, necessitating culturally grounded implementation [38,76,77]. Co-design processes with communities are essential to ensure that repertoire selection, timbral textures, and social formats resonate emotionally and are acceptable to participants [76,78]. Outcome measures must extend beyond standardized clinical metrics to include locally meaningful endpoints such as social capital, identity formation, and ritual participation [72,79]. Furthermore, translational strategies must account for local employment in cultural sectors and the availability of community musical infrastructure [75,76,80,81]. Equity considerations are paramount: structural barriers such as cost, transport, and digital divides must be identified and mitigated, while differential effects across socioeconomic strata should be prospectively evaluated and transparently reported [76,82,83]. These steps respect music’s cultural functions, from work songs and ritual chant to contemporary protest genres, while ensuring that interventions do not impose culturally alien models [38,75,76].

#### 3.4.3. Sleep and Circadian Modulation

Music could serve as a targeted, non-pharmacological adjunct for sleep improvement when aligned with circadian physiology and individualized preferences [59,60]. Evidence suggests that slow tempos around 60–80 bpm, low spectral arousal, and familiar calming material support parasympathetic activation and sleep onset [9,10,61]. Translational success depends on careful attention to timing, with delivery during pre-sleep routines or consistent bedtime windows proving most effective [59,64]. Personalization algorithms that incorporate individual preferences could maximize adherence and effect size, while objective monitoring through wearables and ecological momentary assessment may capture sleep latency, efficiency, and subjective quality [60,64]. Protocols should specify session length, timing relative to bedtime, and progression rules for chronic insomnia applications [59,61]. Aligning acoustic design with circadian principles increases plausibility and real-world impact while preserving low cost and scalability [9,60].

#### 3.4.4. Sport and Motor Rehabilitation

Rhythmic auditory stimulation exemplifies how tempo, beat regularity, and predictability could produce rapid sensorimotor entrainment and durable motor plasticity [8,23,84]. Applications such as gait training in Parkinson’s disease illustrate strong translational potential [62,85,86]. Effective implementation requires specification of tempo ranges, cueing schedules, and progression criteria tied to functional benchmarks such as stride length or gait speed [87,88]. Clinician training is essential to equip physiotherapists and rehabilitation teams with cue-design skills and patient selection criteria [7,84]. Combining rhythmic cues with multimodal rehabilitation, including balance, strength, and task-specific training, may potentiate transfer to daily function [7,86]. Objective outcome measures, such as motion capture or standardized mobility scales, should be complemented by mechanistic endpoints, including neural oscillatory entrainment, to strengthen causal inference and optimize translation [38,88].

#### 3.4.5. Spirituality, Ritual, and Meaning Making

Ritualized music engages affective and social networks that bolster meaning, identity, and resilience, thereby enhancing adherence and contextual fit [89,90]. Incorporating ritual elements or culturally salient music into therapeutic programs may increase acceptability and sustainment, provided such integration respects participants’ beliefs and avoids instrumentalization [75,91]. Co-creation with community leaders ensures authenticity, while transparency about therapeutic aims protects participant agency [75,76]. Ritual’s capacity to produce synchronized physiological and neural states may be harnessed to augment social bonding and meta-plastic adaptation [6,9]. Recognizing music’s spiritual roles broadens intervention targets beyond symptom reduction to encompass well-being, sense-making, and communal resilience [72,75].

## 4. Discussion

### 4.1. Summary

Our results synthesize convergent mechanistic and clinical evidence supporting a multisystem cascade by which specified musical inputs (tempo, beat regularity, timbre, predictability, engagement format, dose, and social context) engage neural entrainment and predictive-reward dynamics, modulate oscillatory coupling and large-scale network connectivity, and alter autonomic-neuroendocrine states; repeated, socially reinforced practice then biases metaplastic readiness and may contribute to synaptic and structural adaptations that could relate to symptomatic improvements across depression, anxiety/procedural stress, schizophrenia, mild cognitive impairment (MCI), dementia, neurodevelopmental disorders, sleep disorders, and motor rehabilitation. The strongest randomized evidence currently supports symptomatic benefits (adjunctive music therapy for depression; peri-procedural anxiety reduction; gait improvements with rhythmic auditory stimulation), whereas mechanistic linkage to durable structural plasticity is uneven because many studies are small, heterogeneous in acoustic/procedural specification, lack pre-specified mechanistic endpoints (EEG, fMRI, DTI, HRV, endocrine markers), and have limited long-term follow-up. These converging but uneven findings motivate prioritized recommendations: harmonized acoustic and protocol reporting, adequately powered randomized designs with active controls, pre-registered multimodal mechanistic endpoints, culturally sensitive implementation, and longitudinal follow-up to test mediation and durability.

### 4.2. Practical Recommendations and Future Directions

Future translational research should specify core intervention parameters while identifying flexible elements amenable to cultural tailoring [72,75,92]. Hybrid effectiveness–implementation trials are needed to measure both clinical benefit and scalability metrics, including fidelity, cost, and reach [75,93]. Co-design processes with communities and cultural stakeholders should be formalized to ensure cultural congruence and sustained engagement [76,94]. Objective monitoring tools such as wearables and ecological momentary assessment could provide adaptive dosing feedback and real-world evaluation [59,80]. Equity must be prioritized through mapping of socioeconomic barriers, allocation of subsidies, and transparent reporting of differential effects [72,95]. Ethical governance structures should establish consent pathways, data protections, and community oversight to avoid coercion or exploitation [76,96]. Finally, mechanistic and implementation endpoints should be explicitly linked, incorporating neurophysiological and behavioral markers such as oscillatory entrainment and neuroimaging to strengthen causal inference and optimize translation [10,97,98].

In sum, contextual factors are not peripheral but fundamental determinants of whether music-based interventions may implement their mechanistic promise in real-world settings [75,99]. Cultural origins, educational infrastructures, circadian biology, motor entrainment dynamics, and ritualized social practices collectively shape uptake, efficacy, and sustainability, underscoring the need for thoughtful integration of these dimensions into translational design [59,72,84]. Success will depend on embedding therapeutic aims within education systems, tailoring protocols to cultural contexts, aligning interventions with sleep and motor neuroscience principles, and deploying scalable delivery models under rigorous ethical safeguards [2,80,100]. Equally important is the explicit pairing of mechanistic research—including neurophysiology, neuroimaging, and oscillatory dynamics, with implementation science frameworks that specify intervention parameters, incorporate community co-design, and enable objective monitoring [16,97,99]. Such integration will generate replicable, culturally congruent, equitable, and sustainable music interventions capable of delivering meaningful benefits at both individual and population levels [72,75].

### 4.3. Priority-Ranked Research Agenda

(1) Standardize reporting and protocol core set
  Require the minimum acoustic/procedural checklist (Section 2) and a short core protocol template for RCT registration to ensure basic comparability.
(2) Adequately powered, mechanistic RCTs with active controls
  Multi-site trials that prespecify mechanistic endpoints (EEG/MEG oscillations, fMRI connectivity, HRV/cortisol) and use active comparators to isolate music-specific effects.
(3) Pre-registered multimodal mechanistic endpoints and mediation analysis
  Combine neurophysiology, imaging, and peripheral biomarkers with prespecified mediation plans to test causal pathways and estimate effect sizes.
(4) Harmonized dose-optimization and stratified designs
  Dose-finding (session length/frequency) and stratification by age, baseline musical engagement, and clinical phenotype to define target populations and effect heterogeneity.
(5) Hybrid effectiveness–implementation studies with cultural co-design
  Concurrent evaluation of clinical effectiveness, scalability, fidelity, equity, and cultural adaptation using hybrid designs and community co-design methods.

Note that implementing the priority agenda outlined above will reduce redundant effort, increase the robustness of causal inference, and expedite translation into clinical and public-health practice, while ensuring cultural adaptability and equitable access.

### 4.4. Limitations and Recommendations for Future Research

Despite convergent evidence suggesting mechanistic plausibility and preliminary clinical signals across several conditions, the current literature remains constrained by multiple methodological limitations [72,75]. Clinical studies frequently employ heterogeneous interventions and lack standardized reporting of acoustic and protocol parameters, such as tempo, rhythmic regularity, duration, and the distinction between active versus passive engagement [7,16,72]. Many trials are underpowered, rely heavily on subjective outcomes, and often fail to pre-specify neurophysiological endpoints [72,86]. Moreover, outcome measures are inconsistent across studies, and longitudinal imaging or DTI evidence directly linking interventions to structural plasticity is scarce [5,33,62]. These limitations hinder reproducibility and complicate the interpretation of effect sizes and target populations [75].

Equally important, the absence of rigorous mechanistic endpoints, such as EEG oscillatory dynamics, fMRI functional connectivity, DTI-based tract metrics, and autonomic or endocrine measures, limits the ability to move beyond associative findings toward causal inference [10,16]. Overly broad claims regarding disease modification are therefore premature, and realistic expectations must acknowledge that current evidence is insufficient to establish durable structural or functional change [5,16,72]. To advance the field, future research should prioritize adequately powered randomized controlled trials with active control conditions, stratification by age and baseline musical engagement, and long-term follow-up to assess durability and structural outcomes [5,7,62]. In addition, culturally tailored protocols should be integrated, with systematic evaluation of adherence and social contextual moderators, to ensure both ecological validity and equity in clinical application [72,101,102]. Mechanistic trials with careful design will help determine whether promising associations can be developed into clinical interventions, provide estimates of effect size, and clarify appropriate target populations [16,103].

## 5. Conclusions

Music exerts multifaceted, mechanistically plausible effects on mental health by engaging interacting mechanisms across neural, autonomic, endocrine, and social systems [10,16]. Acoustic features drive neural entrainment and prediction-reward signaling, which converge with autonomic and neurochemical processes to create an environment conducive to synaptic and structural plasticity [5,6]. When acoustic parameters, engagement formats, and social contexts are deliberately specified and aligned with mechanistic hypotheses, music-based interventions may harness these neuroplastic processes to ameliorate symptoms, foster recovery, and promote resilience across diverse psychiatric and neurological domains [7,66]. Robust clinical translation, however, requires harmonized intervention specification, standardized reporting of acoustic and protocol parameters, and the pre-registration of mechanistic endpoints supported by multimodal measurement strategies [16]. Adequately powered randomized controlled trials with active control conditions, stratification by age and baseline musical engagement, and long-term follow-up are essential to establish durability and structural change [5,62]. Interdisciplinary collaboration, bridging neuroscience, clinical psychiatry, rehabilitation sciences, ethnomusicology, and implementation science, will be critical to accelerate the development of culturally sensitive, scalable, and contextually tailored music-based strategies that complement biomedical approaches and expand accessible pathways for mental-health promotion [16,73,75,99].

## Figures and Tables

**Table 1:** Overview of Evaluation Summaries on the Effects of Music Therapy for Major Mental Health Disorders.

Clinical Domain	Typical Study Designs	Typical Sample Size Range	Key Outcomes Reported	Evidence Quality (High/Moderate/Low/Very low)	Main Risk-of-Bias/Limitations
Depression	RCTs; individual music therapy trials; meta-analyses	n ≈ 30–200 per trial; pooled meta-analytic samples larger	Depression scales (HAM-D, BDI); mood; social functioning; few mechanistic endpoints (EEG/fMRI rare)	Moderate	Heterogeneous interventions; variable acoustic reporting; occasional lack of active controls; limited mechanistic endpoints; variable blinding
Anxiety/procedural stress	RCTs (peri-procedural); smaller trials for chronic anxiety; systematic reviews	n ≈ 20–200	State anxiety scales; Heart rate; Blood pressure; sometimes cortisol/HRV	Moderate (procedural); Low (chronic anxiety)	Short follow-up; small samples for chronic anxiety; heterogeneous comparators; infrequent standardized HRV/cortisol sampling
Neurodevelopmental disorders (ASD, ADHD)	Small RCTs; controlled observational studies; systematic reviews	n often <50 per arm	Joint attention, imitation, social reciprocity (ASD); attention tasks, behavioral ratings (ADHD)	Low–Moderate	Small sample sizes; diagnostic heterogeneity; limited longitudinal neuroimaging; variable intervention protocols
Schizophrenia	RCTs; group and individual therapy trials; Cochrane/systematic reviews	n ≈ 30–150	Negative symptoms; social functioning; Quality of life; limited mechanistic imaging	Moderate	Heterogeneity of adjunctive treatments; variable control conditions; limited mechanistic endpoint reporting; outcome assessor blinding inconsistently reported
Mild cognitive impairment (MCI)/dementia/	RCTs; cluster trials; systematic reviews/meta-analyses	n ≈ 20–150	Agitation; engagement; behavioral/psychological symptoms; transient verbal fluency/attention effects; few structural imaging endpoints	Low–Moderate (symptomatic); Low (structural modification)	Reliance on subjective outcomes; short follow-up; inconsistent imaging endpoints; variable intervention standardization

Notes on table construction and interpretation: Evidence quality judgments reflect a pragmatic synthesis of design robustness, sample sizes, consistency of effects, directness of mechanistic endpoints (EEG/fMRI/DTI/HRV/endocrine), and identified risk-of-bias concerns; they are conservative to avoid overstatement of mechanistic claims. The table summarizes domain-typical findings rather than exhaustive study-level appraisal; exemplar primary trials and systematic reviews cited in the manuscript ([11,18,20,39,41,42,43,44,47,50,67,68,69]) underpin the domain summaries and the table.

## Data Availability

No new data were created or analyzed in this study.

## Abbreviations

The following abbreviations are used in this manuscript:

ADHD	Attention-deficit/hyperactivity disorder
ASD	Autism spectrum disorder
bpm	beats per minute
DTI	diffusion tensor imaging
EEG	Electroencephalography
fMRI	functional magnetic resonance imaging
HRV	heart rate variability
MCI	mild cognitive impairment
MEG	magnetoencephalography
PET	positron emission tomography
RCTs	randomized controlled trials
